# Promotion of Health-Harming Products on Instagram: Characterizing Strategies Boosting Audience Engagement with Cigar Marketing Messages

**DOI:** 10.3390/ijerph22081285

**Published:** 2025-08-17

**Authors:** Ganna Kostygina, Hy Tran, Chandler C. Carter, Sherry L. Emery

**Affiliations:** Ganna Kostygina, Principal Research Scientist, Social Data Collaboratory, NORC at the University of Chicago, 55 E Monroe Street, 30th Floor, Chicago, IL 60603, USAcarter-chandler@norc.org (C.C.C.); emery-sherry@norc.org (S.L.E.)

**Keywords:** social media, advertising and promotion, health communication, cigar, tobacco industry, commercial determinants of health

## Abstract

Social media promotion of harmful products (e.g., combustible tobacco) poses a public health threat. However, strategies that amplify exposure to and engagement with such content remain understudied. This study aims to characterize strategies boosting cigar, little cigar, and cigarillo (CLCC) marketing visibility, referrals, and engagement on Instagram. Using keyword rules, we collected publicly available CLCC-related Instagram posts from CrowdTangle for a six-year period from August 2016 to October 2021. Posts were categorized as commercial (e.g., posts by tobacco brands or vendors) or organic and were coded for consumer engagement (CE) strategies (e.g., presence of prompts to like/share) using a combination of machine learning methods and human coding. Temporal engagement trends were analyzed using metadata. A total of 320,488 CLCC-related public posts were collected, with 44.6% (*n* = 142,875) identified as overtly commercial. Of these, 33.5% (*n* = 47,832) contained CE cues, including discounts and giveaways for tagging peers, liking, commenting, or following CLCC brands and spokesperson/influencers accounts, as well as calls to participate in contests and polls. Overtly commercial CE messages consistently garnered more comments per post and likes per post than non-CE commercial posts. There was a significant upward trend in the rate of comments on CE posts, suggesting growing effectiveness in eliciting user interaction. The proliferation of and high level of engagement with cigar-related promotional messages on Instagram demonstrate the need for public health surveillance and regulation of the evolving strategies promoting CLCC marketing exposure, reach, and engagement on social media.

## 1. Introduction

Disease prevention and health promotion sciences often lag behind the rapid changes in the commercial product landscape and the evolving media environment. Similarly, regulatory structures that protect audiences targeted by marketing of products that are harmful to health have not kept up with the way marketing happens on digital media [1]. The promotion of one of the most important commercial determinants of health—tobacco products—on social media is a prime example of how novel and unregulated marketing creates significant risk factors for premature deaths and imposes costs on individuals and the healthcare system in the trillions of dollars [2]. The seemingly sudden emergence and popularity of JUUL e-cigarettes and Zyn nicotine pouches in recent years illustrate these points [3,4]. The epidemic growth in JUUL and Zyn sales and youth uptake of e-cigarette and nicotine pouch use has been accompanied by an exponential growth in social media messaging, driven by sophisticated marketing tactics enhancing message dissemination and reach [3,4,5].

Instagram represents a particularly important platform for examining health-harming product promotion due to its unique characteristics. Unlike text-based platforms like X (formerly Twitter), Reddit, or Facebook, or professional networks like LinkedIn, Instagram’s “visual-centric” format enables sophisticated multimedia marketing strategies that can seamlessly integrate commercial messages with lifestyle content and provide tobacco marketers with direct access to populations across sociocultural and demographic backgrounds [6,7,8]. Instagram’s algorithmic features that prioritize engagement-driven content create a particularly conducive environment for viral marketing strategies, as posts generating high levels of likes, comments, and shares receive increased visibility and reach [1,7,9].

While recent trends in digital promotion of newer products like e-cigarettes—battery-powered devices that deliver nicotine through vaporized liquid—are well-documented, marketing strategies used to promote combustible non-cigarette products, such as cigars, little cigars, and cigarillos (CLCCs), on social media, including Instagram, are understudied [10,11,12].

In the United States, CLCCs are a leading combusted tobacco product used by youth, with 330,000 (1.2%) U.S. middle and high school students reporting current CLCC use in 2024 (in comparison, the prevalence of cigarette use is 1.4%) [12] and a noticeable rise in initiation as youth transition to young adulthood. In fact, nearly one-quarter of U.S. young adults (23.1%) report having used CLCCs by age 25 [13]. CLCCs are also one of the most discussed tobacco product categories on social media. Prior research shows that the volume of CLCC-related posts exceeds that of cigarettes and smokeless tobacco discussion on platforms such as X/Twitter and Instagram [14,15]. The CLCC market is expected to grow at an annual rate of 7.4% from 2024 to 2030 in the United States [16].

### 1.1. Social Media Marketing Policies

Social media platforms have large youth audiences, as platform policies typically allow adolescents aged 13 and over to open accounts [17]. While the U.S. Food and Drug Administration (FDA) has general authority over tobacco marketing that extends to social media, it has yet to exercise this authority beyond very narrow applications. The 2009 Family Smoking Prevention and Tobacco Control Act (FSPTCA) and the 2016 “Deeming Rule” include prohibitions on the sale and marketing of tobacco products to minors and the distribution of free samples [18,19]. The 2009 FSPTCA cigarette marketing restrictions, which include bans on youth-appealing marketing (e.g., promotion in outlets with large youth audiences), set a precedent that could be applied to other tobacco products and social media promotion [20].

Although in April 2019 the FDA banned paid digital product marketing and influencer promotion of newly introduced tobacco products (such as the IQOS heated tobacco brand), digital promotion of other non-cigarette tobacco products, such as CLCCs, continues to be unregulated [21,22]. Further, bans on disclosed paid advertising capture only a fraction of the tobacco marketing on social media and do not account for strategies like product placement or paid mentions [23], which fall under the category of “covert advertising,” i.e., advertising that intentionally obscures its persuasive intent [24]. While traditional media have featured covert advertising on television and in films, social media platforms introduce new dynamics. On social media, promotional content is seamlessly interwoven with organic discourse. This seamless integration can distort perceptions of product safety and social norms, especially when amplified by algorithmic curation and peer-to-peer sharing [25,26].

Thus, the proliferation of covert promotional practices on social media platforms raises significant ethical concerns about commercial manipulation of information and social responsibility. These strategies exploit users’ trust by leveraging perceived authenticity by featuring content that appears as genuine peer recommendations rather than commercial advertising, thereby lowering the audience’s critical evaluation barriers [1].

Most social media platforms prohibit paid tobacco advertisements; however, enforcement of these policies varies across platforms and product categories [1,21,23,27]. In addition, neither regulatory agencies nor social media platforms limit tobacco brands’ or vendors’ abilities to promote their products via their official social media accounts.

Covert advertising practices, compounded by the absence of specific regulations, create a social responsibility vacuum, in which platforms, brands, and influencers prioritize engagement-driven monetization over public health considerations, with particular risks to vulnerable populations, including youth and at-risk communities who disproportionately use these platforms [28,29]. As a result, social media promotion can encourage the uptake and use of harmful products such as CLCCs, which not only amplifies individual health risks but also imposes costs on the healthcare system [30,31,32].

### 1.2. CLCC Viral Marketing Strategies

As a result, CLCC products continue to be marketed on social media using sophisticated strategies aimed at boosting the reach and virality of branded content, for instance, through influencer promotion and by incentivizing audience engagement with commercial messages [33,34]. CLCC brands, influencers who (formally or informally) represent them, and vendors of CLCC products rely on user engagement to promote their brands and products and, ideally, achieve virality by transforming communication networks into influence networks to amplify visibility, searchability, and word-of-mouth discussions of brand-related content [25,26,35,36,37,38]. Thus, viral marketing may be defined as the type of promotion that relies on the audience to organically propagate the message of a product via peer-to-peer networks [7]. The fact that such marketing takes advantage of a confluence of sophisticated interactive messaging and popular culture makes it particularly effective in gaining traction among the target audience [39].

Viral marketing tactics help drive *“referral contagion”* among social media users, i.e., the sharing of positive experiences and endorsements of brands within individuals’ personal or professional networks [39]. Such engagement is achieved when brands, influencers, or vendors offer overt incentives for content creation and sharing (e.g., discounts, sweepstakes, giveaways, rewards for tagging others, posting response/retweet, following/subscribing) or prompt message sharing by incorporating “shareable” social media content, such as memes, jokes, popular culture references (music, computer games, sports references), or hashtags [14,40,41]. By responding to these calls to action or cues, individuals who interact with the brand, retailer, or influencer become brand ambassadors within their own social networks. A particular challenge with such viral marketing strategies is that they leverage the social networks of the influencers and regular people who engage with either the brand or influencer account, making the marketing highly visible to pockets of consumers, yet entirely invisible to others [42,43]. Viral marketing thus enables users to create “filter bubbles” and “echo chambers”, in which CLCC use is normalized and integrated into social activities, cultural events, environments, or “lifestyles” [44,45].

### 1.3. CLCC Promotion on Instagram

Prior research has shown that CLCC-related conversation and influencer marketing are proliferating on X/Twitter [14,46,47], with nearly 1.5 million messages posted on CLCC products per month, 23% of which were posted by influencer accounts with over 1000 followers, including community accounts (e.g., rap-lifestyle communities) [14]. However, research on CLCC-related discussion on Instagram is limited [48]. Instagram is especially popular among youth and minoritized populations [28,29,49]. According to a 2023 report by the Pew Research Center, 59% of American youth (ages 13 to 17) reported using Instagram [28]. In addition, approximately 49% of Blacks and 52% of Hispanics are Instagram users [50]. In fact, youth, African Americans, and Latinos generally use social media at higher rates than the general population in the U.S. [29,50,51]. Therefore, Instagram appears to be an ideal platform for examining CLCC-related conversation and marketing strategies targeting these populations, as these groups are traditionally at higher risk for CLCCs and are hard to reach using other media channels. Yet, to the best of our knowledge, to date, no studies have systematically explored the quantity and themes of Instagram messages promoting the virality and reach of tobacco marketing content.

The objective of the present study is to fill this gap by analyzing the amount, content, and engagement with CLCC-related Instagram posts. Specifically, we analyzed Instagram messages to identify themes of CLCC content, as well as the volume of and engagement with relevant Instagram posts over time. We used the message content and related metadata to investigate the marketing strategies used by the industry to enhance the engagement and reach of messages promoting CLCC products, with a specific focus on overt calls to engage (like, share, tag friends, etc.) with promotional messages and the use of incentives for content creation and sharing.

## 2. Materials and Methods

### 2.1. Data Acquisition

Hashtag-based keyword rules were used to collect CLCC-related English-language posts for the period from August 2016 to October 2021 from CrowdTangle—a licensed Instagram data provider (now defunct) that collected accounts representing public discourse, including all verified users, celebrities, athletes, sports teams, politicians, media and publishers, and public figures and entities, excluding “regular” or private Instagram user activity. The 5-year study period helped capture a significant period of the CLCC marketing evolution on Instagram when comprehensive data collection was feasible. This time frame represents a critical period in which social media marketing strategies were rapidly evolving and tobacco companies were increasingly sophisticated in their digital promotion tactics [23,52]. More recent data collection has become increasingly challenging due to the social media industry’s (including Meta’s) progressive restrictions on data availability following policy changes and increased scrutiny of social media data access [53,54]. CrowdTangle, our data source, was discontinued in 2024, making this dataset particularly valuable for longitudinal analysis of tobacco marketing trends [54].

We used CrowdTangle to retrieve public social media posts matching one or more search rules in the body of the primary Instagram post. Comments or replies to the primary post could not be matched against the search rules and thus are not included in the total message volume count.

To identify potentially relevant terms and hashtags, we used prior literature and research team expert consensus based on knowledge of CLCC-related terminology and brands. Sample hashtag search terms included cigar, cigars, whiteowl, dutchmasters, cigarillos, swishers, cigarillo, swishersweet, captainblack, blackandmild, rillo, whiteowls, rillos, hoodwraps, garciayvega, swishergang, rellos, blackandmilds, captainblacks, swisherracing, milds, philliesblunt, swishersweeties, swishersonly, havatampa, swisherlife, swisherblunts, littlecigar, swisherssweets, littlecigars, swishersbythecases, swishersweetie, dutchmasterscigars, etc.

Data Cleaning: The retrieved data were cleaned using a combination of human coding and supervised machine learning methods [55]. Specifically, we assessed the quality and validity of the data using rigorous procedures developed by our team (e.g., [56] and others [57,58,59]). Two trained coders rated a random sample of 2273 primary posts (stratified by month of data collection) for CLCC relevance based on the visual and language components of each post, achieving high agreement (α = 0.93) on an overlap sample of 100 posts. Posts that were largely in non-English languages were excluded from analyses. Since our data collection focused on English-language posts, content from various English-speaking countries beyond the U.S. may also have been included. To assess geographic scope, two coders labeled a stratified random sample of 400 posts to assess geolocation (α = 0.88) and found that approximately 78% appeared to originate from U.S.-based accounts based on account information, hashtags, and contextual cues. However, we acknowledge that social media content can transcend geographic boundaries, and some international posts may be included in our dataset.

The text and metadata associated with each labeled post were used to train the machine learning classifier to distinguish the CLCC-relevant posts from irrelevant posts. The linear support vector machine (SVM) classifier with L1-norm regularization was selected via grid search due to its high performance. Tenfold cross-validation was utilized to assess the accuracy of the classifier [60]. The relevance classifier demonstrated 92% accuracy; 95% recall (sensitivity); and 94% precision (positive predictive value) (F1 = 0.95).

### 2.2. Content Coding

Similarly, supervised machine learning was used to classify all retrieved primary posts based on consumer engagement content. Thus, we classified relevant posts as those encouraging engagement (likes, follows, replies) and dissemination (shares, tags), and those without such cues. Sample search rules were “shout AND out(s), s/o, ambassador(s), spokesperson, tag(s), follow(s), contest, loyal, supporter*, participa*”.

Resultant codes were used to train the linear SVM classifier. Classifier accuracy was 83%; classifier recall (sensitivity) was 79%; and precision was 83% (F1 = 0.80 via cross-validation).

In addition, we classified retrieved posts as those containing *overtly* commercial, promotional, or sponsored content and those containing non-commercial user content. More specifically, overtly commercial posts were defined by the presence of any of the following: branded promotional messages; URLs linking to commercial websites; and hashtags indicating affiliations with commercial sites. Two human coders rated a sample of 1782 primary posts (inter-coder reliability was high: α = 0.90), and their codes were used to train the linear SVM classifier. The commercial content classifier demonstrated 80% accuracy, 80% recall (sensitivity), and 80% precision (F1 = 0.80 via cross-validation). While the remaining posts did not feature overtly commercial or direct marketing cues, they were limited to publicly available messages from verified users, celebrities, athletes, sports teams, politicians, media and publishers, and public figures and entities. These posts excluded regular or private Instagram user activity. This content was labeled as “non-commercial,” although it could not be characterized as “organic”, as such messages could potentially contain indirect promotion of CLCC products by influential users.

### 2.3. Metadata

In addition to content or body, each primary Instagram post contains metadata–data about the data, including embedded information about the number of individuals who liked the post, the number of comments the message garnered, the hashtags used to place the message in the context of a broader conversation, post language, etc. Metadata allow inferences about message origin, the number of users potentially exposed to that content, and message engagement. Thus, metadata were used to characterize the source, reach, and impact of CLCC-related posts.

### 2.4. Trend Analysis

The Mann–Kendall trend test was used to detect significant trends in the volume of posts, comments per post, and likes per post, overall and by post type (e.g., trends in the number of posts featuring consumer engagement cues and posts that did not include consumer engagement cues) [61].

Ethical approval for all research activities for the present study was obtained from NORC at the University of Chicago’s Institutional Review Board (IRB #200818).

## 3. Results

**Figure 1:**
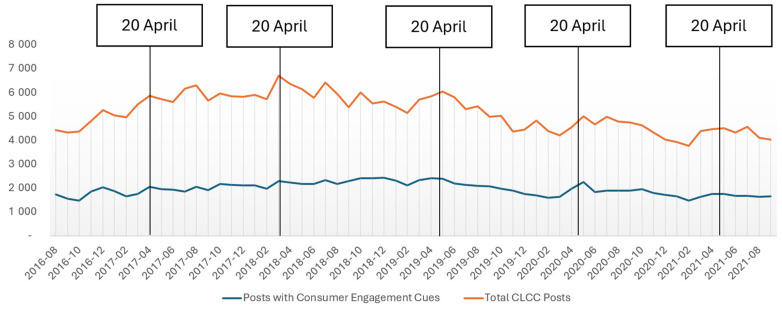
Number of all publicly available cigar-related posts and posts featuring consumer engagement cues over time.

Our list of hashtag-based keywords captured 320,488 CLCC-related public primary posts/conversations posted by 30,007 unique accounts over the study period from 1 August 2016 to 31 October 2021. As illustrated in Figure 1, the temporal trends show distinct patterns in CLCC-related posting activity. Thus, there was a decreasing trend in the number of CLCC posts over time (slope = −31.6; intercept = 6030.0; *p*-value < 0.001), with notable small spikes around 20 April (or “4/20” in the U.S.)—a date associated with cannabis culture that appears to drive cross-promotion of tobacco products—every year during the period of observation (Figure 1).

A large proportion of CLCC-related primary posts were labeled as overtly commercial: about 23% of users (*n* = 6815) posted overtly commercial messages, with the total number of overtly commercial posts being 142,875, or 44.6% of all posts. In addition, we used metadata to assess engagement with CLCC-related posts (i.e., the number of primary post “likes”) over time. The findings revealed that over 38% of primary posts (122,165 messages) featured calls for consumer engagement, with only 254 (<1%) posts featuring a disclaimer disclosing brand partnership or sponsorship. Posts featuring consumer engagement exhibited a decreasing trend over time (slope = −5.4; intercept = 2122.0; *p*-value = 0.022), with posts without consumer engagement cues experiencing a sharper decline (slope = −24.4; intercept = 3860.0; *p*-value < 0.001).

Commercial posts promoting virality featured discounts and giveaways for tagging friends/peers, liking, commenting, or following the CLCC brand and spokesperson/influencer accounts, and included calls to participate in contests and polls (e.g., asking viewers to select their preferred product flavor). For instance, a promotional post by the Good Times brand (@smokegoodtimes) incentivized engagement by featuring a call to action to *“Make sure to Like and Follow in order to have a chance to win $250 gift card!”* In another sample post, @weknowsmokeshop encouraged viewers to *“tag someone you want to give these small batch Backwoods to!! This giveaway winner will be someone you tagged in the comments. Winner will be announced this Friday. Good luck everyone FOLLOW US WE’LL FOLLOW YOU TOO!”*.

**Figure 2:**
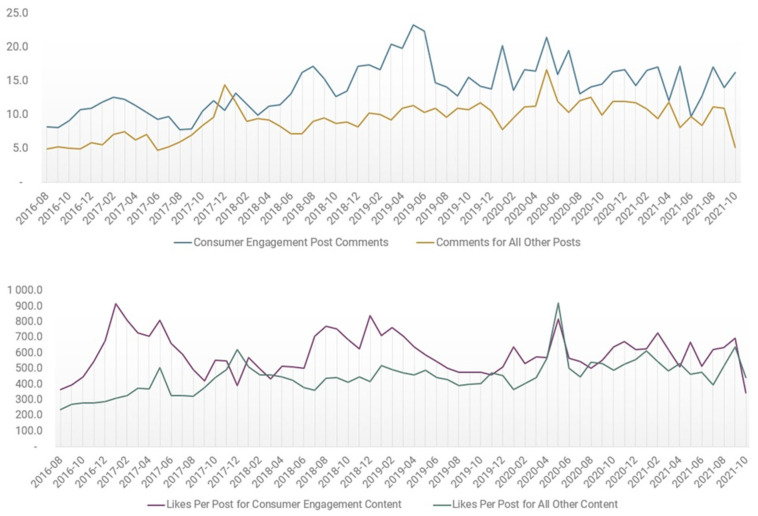
Comments per post (**top**) and likes per post (**bottom**): cigar posts featuring consumer engagement cues versus all other cigar-related posts over time.

CLCC posts featuring consumer engagement calls generated consistently higher numbers of comments and likes per post than posts without such cues during the six-year study period (Figure 2).

Specifically, commercial messages containing engagement calls garnered more comments per post (11) and likes per post (403) on average than commercial posts that did not include such cues (seven comments and 372 likes per post on average). Over time, comments per post significantly increased among consumer engagement posts (slope = 0.12; intercept = 10.3; *p*-value < 0.001), with likes per post among consumer engagement messages remaining relatively stable, with no significant change in volume trend (slope = 0.22.4; intercept = 568.0; *p*-value = 0.803)

The accounts generating the highest engagement were global and platform-native celebrities (popular actors, sponsored musicians/DJs), CLCC- and cannabis-related communities/lifestyle accounts (e.g., @girlswithcigars, @cigaroftheday, @highmerica), and accounts promoting cigar brands and accessories (e.g., @xikar) and alcohol products (Table 1).

The most frequently mentioned Instagram accounts included cannabis-related commercial and community accounts, CLCC brand accounts, cigar community accounts, alcohol-vendor accounts, and a culture/poetry account.

The most frequently mentioned hashtags are presented in Table 2 and predominantly included cigar and cigar-related terms in overtly commercial posts and cannabis or cannabis community-related terms in non-commercial posts.

## 4. Discussion

This study is the first to quantify and characterize the presence of marketing messages that use proactive consumer engagement strategies and incentives to promote CLCCs. Our findings demonstrate that Instagram has historically served as a largely unregulated channel for CLCC promotion, with commercial accounts consistently using viral marketing tactics to boost the engagement with and reach of messages marketing CLCC brands. The present study builds on a growing body of literature that analyzes the promotion of CLCC products on social media platforms, such as X/Twitter, YouTube, and Instagram [14,47,48,62], by documenting marketing strategies, including contests, giveaways, and peer-tagging incentives, that are used effectively by cigar-product vendors and brands to transform social networks into vehicles for cigar promotion on Instagram.

Specifically, the results of our analyses indicate that tobacco companies, brands, and vendors actively use Instagram for consumer engagement through proactive strategies that promote audience participation, such as polls, brand-ambassador recruitment, sweepstakes, contests, and frequent shout-outs to consumers. Over the course of the study period, the number of overt user engagement posts featuring viral cues (e.g., calls to like, follow, or tag a friend) constituted approximately 33.5% of conversations. Thus, Instagram provides an opportunity for commercial and influential users to foster relationships with customers and integrate their brands and imagery into users’ organic social networks.

The significantly higher rates of likes and comments for posts containing consumer engagement cues suggest that these strategies effectively amplify tobacco messaging. While the number of consumer engagement posts exhibited a slight decline across the study period, the rate of comments on such posts increased significantly. The decreasing trend in posts promoting engagement with CLCC marketing over our study period likely reflects multiple converging factors. Platform enforcement of tobacco-related content policies may have reduced overt promotional activity, while simultaneously driving a shift toward more sophisticated, harder-to-detect marketing strategies [23]. Additionally, shifts in youth preferences toward platforms like TikTok may have prompted marketers to diversify their social media presence [63]. Importantly, despite the decrease in volume, the significant increase in the rate of comments on consumer engagement posts suggests that the remaining content became more targeted and effective at eliciting user interaction, indicating strategy refinement rather than reduced marketing effectiveness. This is particularly concerning given Instagram’s popularity among minoritized populations, who are already at disproportionate risk of tobacco-related harms [29].

Further, we found that the proportion of overtly commercial posts promoting CLCC use was relatively high on Instagram (44.6% of conversations), with these posts consistently generating a high level of engagement. This finding is consistent with research suggesting that Instagram has been an especially valuable tool for marketers since it gained widespread popularity in the U.S. and globally over a decade ago, with the platform historically effective in delivering engagement and acquiring brand ambassadors [64], including brands and products that are potentially harmful to health [9,65].

Finally, our study contributes to the emerging body of literature on promotion within the commercial determinants of health on social media and the co-promotion of tobacco, cannabis, and alcohol substances in particular [66,67,68]. Thus, our analysis revealed that the most frequently referenced Instagram accounts in the CLCC-related discussions on Instagram included cannabis-related commercial and community accounts, as well as alcohol-vendor accounts. The fact that marketing on cannabis and alcohol-related accounts generated some of the highest engagement levels with CLCC content reveals similarities in the tactics that boost virality across products and demonstrates the coordinated, cross-promotional nature of such marketing. The engagement strategies we documented, transforming users into brand ambassadors through their own personal networks, represent a fundamental shift in how commercial actors promote health-harming products on social media, which appears to extend beyond CLCC marketing alone.

In summary, our study demonstrates that commercial content promoting harmful product use and incentivizing social media users to engage with such content is publicly available and easily accessible. This promotion is present regardless of platform self-regulatory activities [23]. Active use of consumer engagement tactics is particularly concerning since viral social media marketing used by the vendors and manufacturers of harmful commodities, such as CLCCs, can amplify youth and new-user exposure to tobacco content. Our findings indicate that influencer and “community” accounts associated with harmful products beyond tobacco (e.g., cannabis- and alcohol use-related accounts) encourage engagement with CLCC promotion, which helps normalize CLCC use and boost promotional reach.

In the U.S., both the FDA and the Federal Trade Commission require social media influencers to disclose material relationships with brands they represent, yet studies show that as few as 4% of influencer posts disclose such relationships [9,69]. Enforcement of sponsorship disclosures in influencer posts can be an effective deterrent, as they decrease perceptions of influencer credibility and reduce intentions to engage with posts [70]. Future research should identify novel, comprehensive, and enforceable approaches to regulate and counter CLCC and other harmful product marketing on social media.

### Limitations

This study has several limitations. CrowdTangle social media data capture publicly available posts by influential users (i.e., verified users, public figures and entities), which may limit the comprehensiveness of the retrieved data, as it excludes commercial, influencer, and organic/regular user accounts with relatively few followers. Our findings were based on the analysis of publicly available posts and did not include promotion via private brand or vendor accounts on Instagram. Further, because the retrieved posts were limited to publicly available messages from verified users, celebrities, and other public figures and entities, the “non-commercial” messages that did not contain overt promotional cues may have contained indirect marketing of CLCC products by influential users.

The study period (2016–2021) occurred as both the tobacco product market and social media landscape were rapidly expanding; however, our findings are not limited to the study period or the Instagram platform. Further, newer tobacco and nicotine brands and products, such as e-cigarettes and oral nicotine pouches, are gaining popularity, in part, due to social media marketing strategies that encourage brand engagement among young people [3]. Youth and young adults have expanded their use of social media platforms to those defined by highly visual content, influencers, and targeted algorithms, such as TikTok [28]. As of 2024, teen Instagram usage is nearly on par with TikTok [63], yet differences in platform affordances—such as content formats, discovery mechanisms, and engagement features—may lead to varying levels of user interaction with CLCC content and, consequently, to varied implications for marketing tactics and effects on health behavior. Unlike TikTok’s algorithm-driven, short-form video content that can rapidly amplify individual posts, Instagram’s established influencer ecosystem and visual format create sustained relationship-building opportunities between brands and users [6,7,8,39,71]. Yet, the fundamental viral marketing strategies we identified, including peer tagging, contests, and influencer partnerships, can be employed across platforms and product categories, suggesting our findings retain contemporary relevance, despite ongoing platform and regulatory changes. Future research should further examine how promotional tactics adapt across platforms and whether regulatory approaches need platform-specific considerations. While our analysis focuses on English-language content with apparent U.S. origins, the global nature of social media platforms means that these findings have international relevance. Tobacco companies increasingly use global influencer networks [72], while consumers use technological workarounds such as VPNs (virtual private networks) [73,74] to circumvent local marketing regulations imposed either by social media platforms or government jurisdictions (e.g., age verification or blocked content), making national-level policy responses potentially insufficient. The cross-border nature of digital marketing suggests that coordinated international approaches to social media tobacco marketing regulations may be necessary to effectively protect public health across jurisdictions.

## 5. Conclusions

This study represents a comprehensive analysis of marketing tactics used to promote engagement with CLCC-related product content on Instagram. Instagram continues to be a popular yet understudied social media platform. Our analysis of over 320,000 publicly available posts from a 5-year period provides unprecedented longitudinal insights into tobacco marketing trends on a platform heavily used by youth and minoritized populations. Our findings demonstrate that commercial entities actively leverage user engagement to amplify tobacco messaging, potentially increasing youth exposure to normalized tobacco content. The proliferation and high level of engagement with publicly available content promoting CLCC use urgently warrant public health surveillance and strategic communication efforts to reframe perceptions of nicotine addiction and stem the normalization.

This research is critical for establishing a scientific and methodological base for the surveillance and potential regulation of commercial messages that promote tobacco products on social media and for advancing the development of communication science and technology by identifying the potential regulatory targets and practices that raise concerns and need to be addressed. The study makes a significant contribution to our understanding of the specific marketing tactics used to encourage dissemination and boost the reach and exposure of commercial messages related to health-harming products and highlights several critical gaps in current regulatory frameworks. Thus, existing regulations fail to address the sophisticated viral marketing strategies and co-promotion tactics across the commercial determinants of health documented in this study.

While potential regulatory approaches—such as requiring clear sponsorship disclosure for all commercial CLCC-, cannabis-, and alcohol-related content; prohibiting engagement incentives like contests and giveaways; mandating age verification for accounts that post content related to tobacco and other health-harming products; and developing cross-platform regulatory frameworks that address the global, interconnected nature of social media marketing—could be promising, their implementation may face practical challenges. These include enforcement difficulties across decentralized platforms, resistance from stakeholders, and circumvention of age-verification systems. Nonetheless, these measures represent important steps toward mitigating youth exposure to harmful product promotion, and efforts should be made to overcome such challenges.

Our findings can also help inform message design and strategies to boost dissemination (e.g., overt calls to action that encourage viewers to like or share posts) for future counter-marketing campaigns on social media. Importantly, these findings are directly translatable to other areas of communication and behavioral research studying the health implications of social media.

## Figures and Tables

**Table 1:** Commercial and influencer accounts generating the highest levels of engagement.

User Name	Comments	Likes	Post Volume
@highmerica	66,327	5571,596	897
@stoned.creature	31,666	1988,679	1209
@[name_redacted] *	30,217	858,321	215
@cigaroftheday	27,606	1717,151	1265
@resolutioncolorado	26,033	178,578	136
@distinguishedruffian	25,602	515,779	2808
@lifeofluxe	23,176	306,731	497
@xikar	22,625	509,702	656
@indianabourbon	19,021	475,571	770
@[name_redacted] *	18,429	705,260	69

* Influencer account name redacted.

**Table 2:** Most frequently mentioned hashtags in overtly commercial and non-commercial posts.

Commercial Posts		Non-Commercial Posts	
Hashtag	Count	Hashtag	Count
#cigars	96,121	#cigar	60,003
#cigar	83,628	#blunt	42,918
#botl	62,729	#cigars	38,503
#cigarlife	60,611	#420	37,007
#cigaraficionado	54,133	#cannabis	33,732
#sotl	48,885	#weed	29,289
#cigarporn	45,471	#smoke	28,148
#cigarsociety	45,425	#marijuana	27,459
#cigaroftheday	42,460	#stoner	23,423
#cigarsnob	34,667	#kush	23,087

## Data Availability

Data may be obtained from a third party/vendor and are not publicly available. The Instagram data were obtained from CrowdTangle—a social media data provider, content discovery, and monitoring platform.

## Abbreviations

The following abbreviations are used in this manuscript:

CLCCs	Cigars, Little Cigars, and Cigarillos
FDA	Food and Drug Administration
FSPTCA	Family Smoking Prevention and Tobacco Control Act
SVM	Support Vector Machine
IRB	Institutional Review Board
